# Modelling Ser129 Phosphorylation Inhibits Membrane Binding of Pore-Forming Alpha-Synuclein Oligomers

**DOI:** 10.1371/journal.pone.0098906

**Published:** 2014-06-09

**Authors:** Georg Sebastian Nübling, Johannes Levin, Benedikt Bader, Stefan Lorenzl, Andreas Hillmer, Tobias Högen, Frits Kamp, Armin Giese

**Affiliations:** 1 Department of Neurology, Klinikum der Universität München, Ludwig-Maximilians-University, Munich, Germany; 2 Center for Neuropathology and Prion Research, Ludwig-Maximilians-University, Munich, Germany; 3 Department of Palliative Medicine, Klinikum der Universität München, Ludwig-Maximilians-University, Munich, Germany; 4 Endowed Professorship for Interdisciplinary Research in Palliative Care, Institute of Nursing Science and –Practice, Paracelsus Medical University, Salzburg, Austria; 5 Adolf-Butenandt-Institute, Munich, Germany; University of South Florida College of Medicine, United States of America

## Abstract

**Background:**

In several neurodegenerative diseases, hyperphosphorylation at position Ser129 is found in fibrillar deposits of alpha-synuclein (asyn), implying a pathophysiological role of asyn phosphorylation in neurodegeneration. However, recent animal models applying asyn phosphorylation mimics demonstrated a protective effect of phosphorylation. Since metal-ion induced asyn oligomers were identified as a potential neurotoxic aggregate species with membrane pore-forming abilities, the current study was undertaken to determine effects of asyn phosphorylation on oligomer membrane binding.

**Methods:**

We investigated the influence of S129 phosphorylation on interactions of metal-ion induced asyn oligomers with small unilamellar lipid vesicles (SUV) composed of POPC and DPPC applying the phosphorylation mimic asyn129E. Confocal single-particle fluorescence techniques were used to monitor membrane binding at the single-particle level.

**Results:**

Binding of asyn129E monomers to gel-state membranes (DPPC-SUV) is slightly reduced compared to wild-type asyn, while no interactions with membranes in the liquid-crystalline state (POPC-SUV) are seen for both asyn and asyn129E. Conversely, metal-ion induced oligomer formation is markedly increased in asyn129E. Surprisingly, membrane binding to POPC-SUV is nearly absent in Fe^3+^ induced asyn129E oligomers and markedly reduced in Al^3+^ induced oligomers.

**Conclusion:**

The protective effect of pseudophosphorylation seen in animal models may be due to impeded oligomer membrane binding. Phosphorylation at Ser129 may thus have a protective effect against neurotoxic asyn oligomers by preventing oligomer membrane binding and disruption of the cellular electrophysiological equilibrium. Importantly, these findings put a new complexion on experimental pharmaceutical interventions against POLO-2 kinase.

## Introduction

Fibrillar deposits of alpha-synuclein are the pathological hallmark of several neurodegenerative diseases such as Parkinson's disease (PD) and multiple systems atrophy (MSA). In PD, the progressive spreading of Lewy bodies to different brain areas coincides with clinical disease progression [1]. In recent years, there is increasing evidence that smaller alpha-synuclein (asyn) oligomers, which are *en route* to fibril formation, are the key neurotoxic aggregate species [2]–[5]. However, the circumstances leading to the formation of these oligomers as well as their toxic mode of action remains unclear.

Increasing evidence suggests that interactions between asyn and lipid membranes may be essential to asyn function and neurotoxicity [6]–[8]. However, these interactions appear to be complex. Concerning physiological protein function, it was shown that asyn physiologically interacts with vesicles at the presynaptic terminals, regulating vesicle traffic [9], [10]. In asyn monomers, membrane interactions are mediated through KTKEGV repeats at the N-terminus [11]–[13]. Such interactions might be related to the protein's native function, stabilizing lipid packing and reducing membrane fusion [14]. On the other hand, several models have shown a neurotoxic effect of asyn membrane binding [15], [16]. Furthermore, asyn membrane binding has been suggested to favor oligomer formation and subsequent disruption of membrane integrity [17], [18]. To date, it is not fully understood which forms of asyn membrane interactions lead to toxicity, and whether oligomer formation is a result of or a requirement for pathologic membrane interactions. To address this issue, we previously performed experiments using single-particle fluorescence techniques, which allow distinguishing between monomer and oligomer binding to membranes. By this approach, we were able to demonstrate that asyn binding to lipid vesicles inhibits oligomer formation, whereas oligomers show enhanced binding to lipid membranes and may act as membrane pores [6], [7]. These data further substantiate the hypothesis that interactions of asyn oligomers with cellular membranes are the key toxic event in synucleinopathies.

Another ongoing controversy concerns the role of asyn phosphorylation in neurotoxicity. Phosphorylation at Ser129 is abundant in Lewy bodies [19], [20], while the phosphorylation rate of asyn at this position appears to be low in the healthy mammalian brain. To date, both pro- [20]–[22] and anti-aggregatory effects [23], [24] of asyn phosphorylation and phosphorylation-mimicking mutations have been observed *in vitro* and in animal models. However, the role of asyn phosphorylation is not limited to modulating aggregation behavior. Several lines of evidence point towards an inhibiting effect of asyn phosphorylation on membrane binding. For example, in both budding yeast and transgenic C. elegans, binding of asyn to plasma membranes is enhanced for the phosphorylation-deficient S129A mutation, while the phosphorylation mimicking S129D mutation results in decreased membrane binding [16], [25]. However, it is currently unknown how phosphorylation modulates membrane binding, i.e. whether it affects physiological monomer binding and/or membrane interactions of disease-associated asyn oligomers.

The current study aimed at elucidating the influence of phosphorylation on interactions of asyn monomers and potentially toxic oligomers with model lipid membranes. Based on earlier findings from histopathological, epidemiological and experimental studies implicating an involvement of ferric iron in the pathogenesis of synucleinopathies, we have established a model of asyn oligomer formation yielding potentially toxic aggregates in presence of ferric iron [26]–[28]. We demonstrated that physiological concentrations of ferric iron result in the formation of a specific asyn oligomer species even at nanomolar protein concentrations [6]. Furthermore, these oligomers have been shown to interact with lipid membranes and may act as membrane pores, possibly disrupting the transmembranous electrophysiological equilibrium [6]–[8], [29].

Here, we applied single-particle fluorescence techniques to monitor the binding of monomers and metal-ion induced oligomers of recombinant human asyn and the phosphorylation-mimicking mutant asyn129E to small unilamellar lipid vesicles (SUV). SUV were composed of 1-palmitoyl-2-oleoyl-sn-glycero-3-phosphocholine (POPC) or dipalmitoylphosphatidylcholine (DPPC), yielding lipid membranes in the liquid-crystalline or gel state at room temperature, respectively. By using fluorophores with two different excitation wavelengths, fluorescence correlation spectroscopy (FCS) and scanning for intensely fluorescent targets (SIFT) allow for the simultaneous detection of two different particle types, thus enabling us to monitor particle interactions and rare oligomers down to attomolar concentrations [30], [31].

## Materials and Methods

### Expression and fluorescence labeling of recombinant human alpha-synuclein and pseudophosphorylation mutants S129D/E

Recombinant human alpha-synuclein was expressed in E.coli and purified as described previously [32]. A stock solution of 1 mg/ml was prepared in tris buffer pH 7.0, 50 mM.

alpha-Synuclein pseudophosphorylation mutants S129E were a kind gift of Prof. Dr. Philipp Kahle (Hertie Institut für klinische Hirnforschung, Tübingen, Germany).

### Fluorescence labeling

Human recombinant proteins asyn and asyn129E were fluorescently labeled with Alexa-647-O-succinimidylester as described previously [32]. Labeling ratios of dye to protein were 2∶1.

### Generation of small unilamellar lipid vesicles

Small unilamellar vesicles (SUV) were prepared from 1-palmitoyl-2-oleoyl-sn-glycero-3-phosphocholine (POPC) or dipalmitoylphosphatidylcholine (DPPC) as described previously [7]. In brief, a fluorescent lipid dye (Bodipy-PE, Invitrogen, Carlsbad, CA) was mixed with POPC or DPPC in chloroform. Lipids were dried by nitrogen gas evaporation, followed by hydration in buffer and sonication. A vesicle diameter of 25–35 nm was confirmed by dynamic light scattering (High Performance Particles Sizer, Malvern Instruments, Herrenberg, Germany). SUV stock solutions (lipid concentration: 100 µM) were stored at 4°C and used within 48 hours.

### Aggregation assay

Protein stock solutions were diluted to a final concentration of 10–20 nM (10–20 particles per focus volume) and coincubated with Fe^3+^ or Al^3+^ at a final concentration of 10 µM for one hour in a total sample volume of 20 µl to induce oligomer formation. Subsequently, POPC-SUV or DPPC-SUV then added at a final lipid concentration of 10 µM. SIFT measurements were started after 30 minutes incubation time. Five measurements were performed on four independent samples per measurement group. Measurement time was 10 s. Measurements were carried out on an Insight Reader (Evotec Technologies, Hamburg, Germany) in 384 well glass-bottom sample plates (Greiner Bioplates, Greiner, Solingen, Germany) as described previously [7].

### Analysis of protein aggregation and membrane binding

Protein aggregation was analyzed as described previously using the software packages SIFT-2D and FCSPPEval Version 2.0 (Evotec Technologies, Hamburg, Germany) [33]. In brief, photons per channel were summarized in time intervals (bins) of 40 µs and plotted in a 2D FIDA (fluorescence intensity distribution analysis) histogram. Histograms depicted in this paper contain all photons of all consecutive measurements of one sample. Bin-weighted SIFT aggregation and coaggregation analysis was performed as described previously [33]. A threshold (T) was determined by examining brightness in monomer samples. Oligomer signal was defined as all photons (N) exceeding the threshold (N>T).

To determine oligomer brightness and size, asyn and asyn129E monomer brightness (Q_monomer_) was determined by a 1D FIDA one component fit in a sample without aggregation inducers as described previously [7]. Subsequently, oligomer brightness (Q_oligomer_) was determined in a 1D FIDA two component fit applying the previously determined monomer brightness as fixed variable. Oligomer size (X_oligomer_) was then calculated by X_oligomer_ = Q_oligomer_/Q_monomer_. The oligomer fraction of the protein solution was calculated by FIDA two component fit applying the formula c_2_q_2_/(c_1_q_1_/c_2_q_2_) (c_1/2_ =  monomer/oligomer concentration; q_1/2_  =  monomer/oligomer brightness).

To determine the brightness of vesicles with bound monomers, 2D-FIDA analysis was performed. Therefore, monomer and vesicle brightness were determined as stated above. Subsequently, the brightness and concentration of bicolored particles was determined by a three component 2D FIDA fit with a pre-set particle brightness in both channels for component one (free asyn monomer) and two (vesicle without bound monomers) and in channel one for the third component (fixed vesicle brightness, free fit of brightness for bound asyn). From the resulting brightness and concentration of the third component, the total fluorescence derived from bicolored particles in the red channel (i.e. asyn) was calculated as X_bicolored_ = C_bicolored_*Q_bicolored_.

Protein binding to lipid vesicles was analyzed applying the SIFT-2D software package. Again, monomer brightness was determined, and an artificial neutral point was defined to exclude vesicles and unbound oligomers. Membrane bound oligomers could thus be identified as 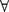
n_bound_∶ x>0 ∩ y>0. Since unbound oligmers are depicted along the ordinate of 2D FIDA histograms, they can be identified as 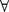
n_ unbound_∶ x<0 ∩ y>0. The percentage of bound oligomers is thus identified as N_bound_/(N_bound_ + N_unbound_).

### Statistical analysis

Normal distribution of data was evaluated by Shapiro-Wilks-Test. Data are displayed as mean ± standard deviation, except for SIFT analyses which are displayed as mean ± standard error of the mean. Levels of significance were determined by student's t-test with Bonferroni adjustment for multiple testing where appropriate. Significance was determined at an α-level of p<0.05. All statistical analyses were performed with the software package SPSS version 20.

## Results

### 1. Pseudophosphorylation at Ser129 mildly reduces asyn monomer binding to lipid vesicles

**Figure 1:**
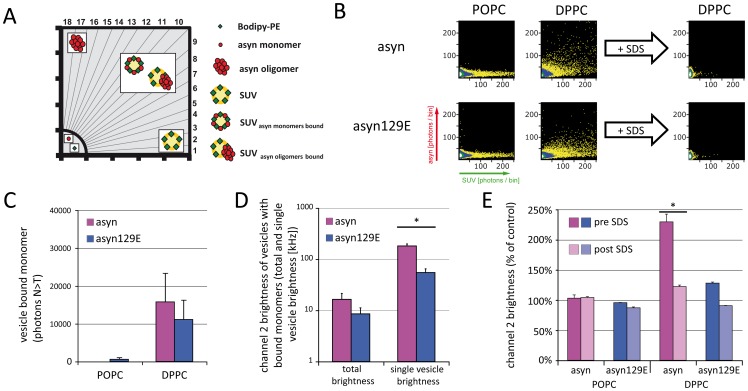
Effect of Ser129 pseudophosphorylation on asyn monomer binding to lipid vesicles. aSyn^647^ and asyn129E^647^ were coincubated with POPC- and DPPC-SUV^488^. Schematic A. demonstrates the appearance of vesicles, protein monomers and oligomers in 2D FIDA histograms (adapted from [7]). B. aSyn^647^ and asyn129E^647^ monomers show no interactions with POPC-SUV. Conversely, extensive monomer binding to DPPC-SUV is seen irrespective of pseudophosphorylation status. C. Quantitative FIDA analysis shows a mild tendency towards reduced vesicle binding of asyn129E^647^. D. Quantitative 2D-FIDA analysis of monomers bound to DPPC-SUV demonstrates an overall higher amount of bound monomers (total brightness), determined through brightness and concentration of bicolored particles. Furthermore, the higher fluorescence intensity of the bicolored particles (single vesicle brightness) indicates that more monomers are bound per vesicle. E. In a control experiment, asyn^647^ and asyn129E^647^ particle brightness (Q) as determined by 1D FIDA analysis (one component fit) is unchanged in presence of POPC-SUV as compared to control measurements in the absence of vesicles, confirming that no membrane binding takes place. However, Q increases in presence of DPPC-SUV. Dissolution of DPPC-SUV by SDS yields an asyn^647^ particle brightness similar to monomeric asyn^647^, indicating that oligomer formation is not induced by binding to DPPC-SUV. Levels of significance are depicted as * = p<0.05; n = 3.

To investigate the influence of pseudophosphorylation at Ser129 on asyn monomer binding to lipid vesicles, fluorescently labeled asyn^647^ and asyn129E^647^ (“red” channel) were coincubated with POPC- and DPPC-SUV^488^ (“green” channel) and measured by SIFT. Data were depicted as 2D FIDA histograms, with monomers resulting in data points near the origin of the histogram, while asyn oligomers and vesicles labeled with multiple fluorophores result in signal along the ordinate and the abscissa of the histogram, respectively. Vesicle-bound monomers and oligomers are represented along the bisectrix of the histogram (also see the schematic in figure 1 A, adapted from [7]).

In the first set of experiments, no interactions of asyn/asyn129E monomers and POPC-SUV were seen, while both asyn and asyn129E readily bound to DPPC-SUV (figure 1 B). To investigate whether binding to DPPC-SUV induces the formation of asyn oligomers, the ionic detergent sodium-dodecyl-sulfate (SDS) was added to the assay after the first set of measurements. After dissolution of lipid vesicles with SDS at a final concentration of 0.2%, only a low-intensity signal remained in both channels, indicating that no SDS-resistant oligomers were formed upon asyn binding to DPPC-SUV. In this experimental setup, asyn129E exhibited a similar pattern of interaction with POPC- and DPPC-SUV as asyn, although a mildly reduced DPPC vesicle binding was seen (see figure 1 C). Fluorescence intensity distribution analysis (FIDA) showed that particle brightness in the red channel (asyn^647^) was unchanged upon coincubation of asyn and POPC-SUV, while an approximately twofold increase in particle brightness was seen upon coincubation with DPPC-SUV (figure 1 E). Furthermore, 2D-FIDA analysis demonstrated that the total brightness of vesicle-bound monomers is mildly higher for asyn as compared to asyn129E (figure 1 D). In this analysis, monomer and vesicle brightness are first determined in control samples. In a second step, these parameters are used to calculate the brightness and concentration of bicolored particles. The fitted single-particle brightness was also higher for asyn, demonstrating that bicolored particles contain on average more asyn monomers as compared to asyn129E (figure 1 D, *single vesicle brightness*). Further indicative of the binding of multiple asyn monomers to one vesicle, particle brightness was reduced to control levels upon coincubation with SDS (figure 1 E), which readily dissolves DPPC-SUV, thus liberating bound monomers. Of note, no asyn oligomers are found after dissolution of DPPC-SUV. A similar, but less pronounced effect was seen for asyn129E.

### 2. Pseudophosphorylation at Ser129 increases metal-ion induced asyn oligomer formation

**Figure 2:**
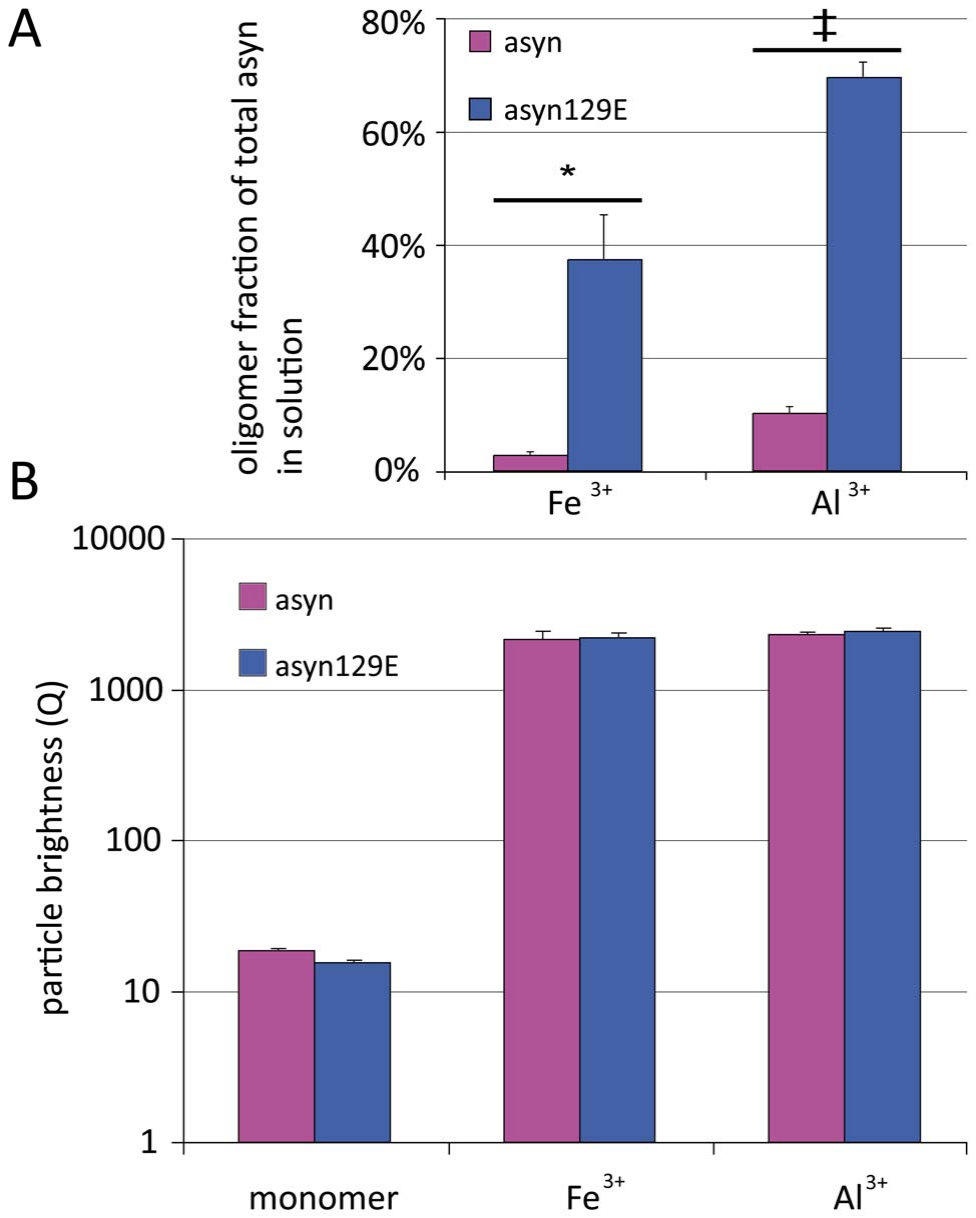
Differential influence of Ser129 pseudophosphorylation on metal-ion induced asyn oligomer formation. A. FIDA analysis demonstrates an increased proportion of asyn129E^647^ oligomers within total fluorescence intensity for both Fe^3+^ and Al^3+^, indicating an increased aggregation propensity of asyn129E^647^ in presence of both aggregation inducers. B. FIDA two component fit demonstrates that oligomer sizes do not differ significantly between asyn and asyn129E. Levels of significance are depicted as * = p<0.05; n = 3.

It was previously shown that physiological concentrations of ferric iron induce the formation of a distinct, SDS-resistant asyn oligomer species [6], [7], [34]. In our experiments, pseudophosphorylation at position Ser129 markedly increased asyn oligomer formation in presence of 10 µM Fe^3+^ and Al^3+^. As determined by 1D-FIDA two component analysis, the fraction of asyn incorporated in oligomers was higher for asyn129E (see figure 2 A). Of note, the presence of ferric iron in concentrations up to 100 µM did not alter the properties of the fluorescence dyes (see figure S1 A, B). Both FCS and FIDA analyses showed that total brightness of the dye in solution, single particle brightness and diffusion time remain unaltered by presence of metal ions (figure S1 A, B, D). In addition, these parameters remained unchanged upon addition of ferric iron to a solution containing a fluorescently labeled non-amyloidogenic protein, in this case the asyn specific antibody 15G7 (figure S1 C). To examine possible effects of random asyn labeling on oligomer formation, a control experiment applying modified asyn proteins with point mutations to cysteine at positions 3, 81 and 140 and subsequent point-specific labeling with Alexa-647 maleimide dyes was performed. In this experiment, monomers of all asyn mutations showed similar FCS signals with comparable diffusion times (figure S1 F). Furthermore, 1D-FIDA analysis of Fe^3+^ induced asyn oligomers yielded comparable oligomer sizes for all labeling sites (figure S1 E), which were well in accordance with oligomer sizes of randomly labeled asyn as presented earlier [35]. Of note, earlier studies applying atomic force microscopy also found similar sizes of metal ion induced oligomers of unlabeled and randomly labeled asyn [6].

Concerning the effect of asyn pseudophosphorylation, FIDA analysis showed an increase in the oligomer fraction in asyn129E for both Fe^3+^ and Al^3+^, indicating an augmented aggregation propensity for asyn129E under these conditions (see figure 2 A). In contrast, oligomer sizes as determined by 1D-FIDA two component fit are not significantly altered by pseudophosphorylation, ranging from 116 to 143 monomers per oligomer for Fe^3+^ and from 125 to 158 monomers per oligomer for Al^3+^ (also see figure 2 B).

### 3. Binding to lipid membranes composed of POPC is a common hallmark of asyn oligomers induced by trivalent metal-ions

**Figure 3:**
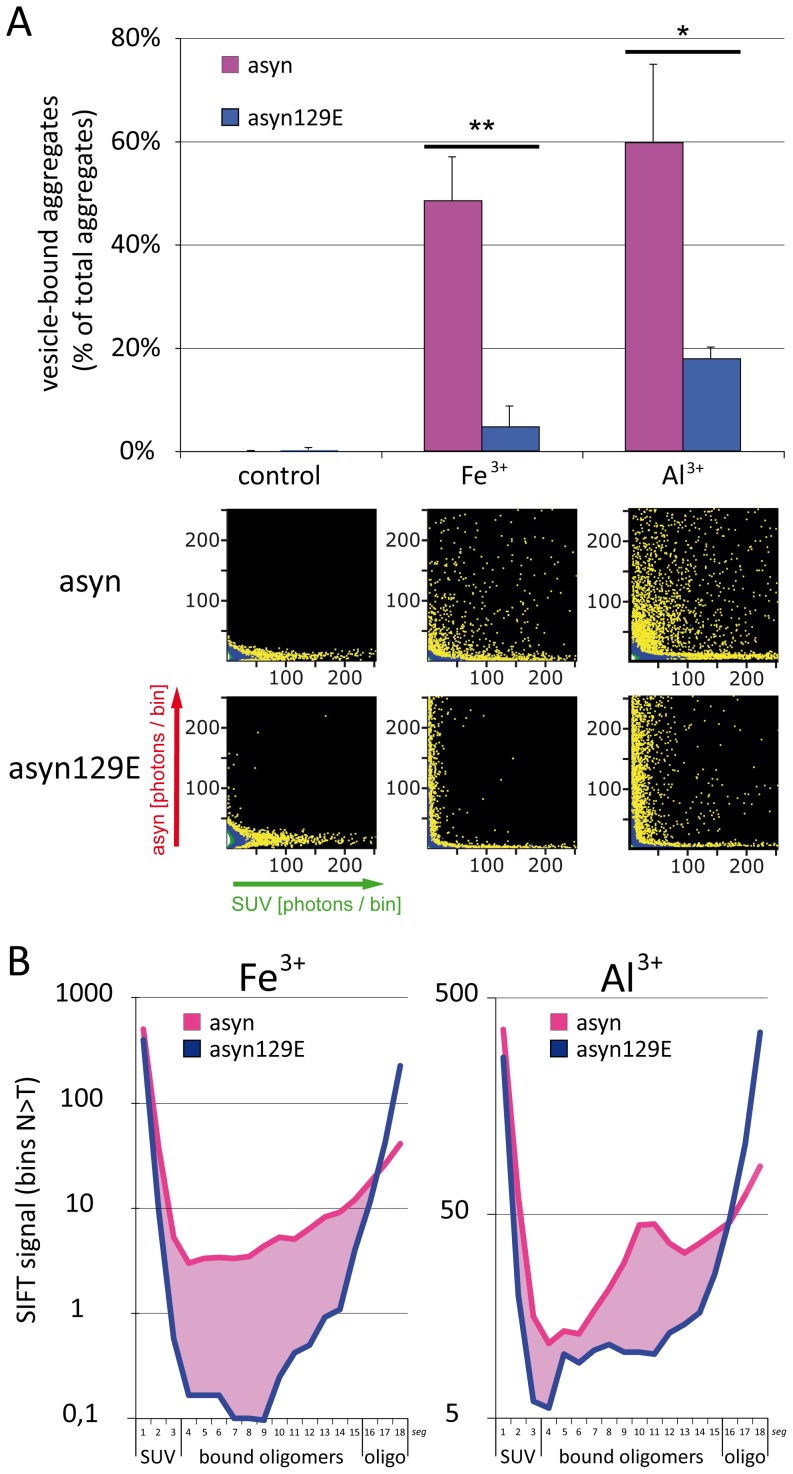
Influence of metal-ion induced asyn oligomer formation and pseudophosphorylation on membrane binding. A. FIDA analysis (upper panel) and 2D FIDA histograms (lower panel) demonstrate that Fe^3+^/Al^3+^ induced asyn^647^ oligomers show a high affinity to POPC-SUV^488^. Conversely, membrane binding of asyn129E^647^ oligomers is nearly abolished for Fe^3+^ and markedly reduced for Al^3+^ induced oligomers. B. SIFT segment analysis also demonstrates an increased mixed particle signal for asyn^647^ as compared to asyn129E^647^ (“bound oligomer”, corresponding to SIFT segments 4–15; also see figure 1A.), while levels of free oligomers are higher in asyn129E^647^ (“oligo”, corresponding to segments 16–18). Levels of significance are depicted as * = p<0.05, ** = p<0.01; n = 3.

We previously reported that Fe^3+^ induced asyn oligomers show a gain of function compared to monomers by binding to SUV composed of POPC. Since Al^3+^ proved to be a more potent inducer of asyn oligomer formation, subsequent experiments were undertaken to compare the binding behavior of asyn oligomers induced by Fe^3+^ and Al^3+^. As is demonstrated in figure 3 A, Al^3+^ induced asyn oligomers showed an overall higher propensity to bind to POPC-SUV as compared to oligomers formed in presence of Fe^3+^ as determined by FIDA analysis.

### 4. Inhibition of asyn oligomer membrane binding by pseudophosphorylation at Ser129

In this set of experiments, we investigated the influence of pseudophosphorylation at Ser129 on oligomer vesicle interactions. While metal ion induced asyn oligomers exhibited strong binding to POPC-SUV, binding of Fe^3+^ induced oligomers was almost abolished (<5% bound oligomers) in asyn129E, and binding of Al^3+^ induced oligomers was markedly reduced (<20% bound oligomers, see figure 3 A). In two control experiments, the size of asyn/asyn129E oligomers formed in presence of POPC-SUV (asyn/asyn129E: 116/134 monomers per oligomer) remained unchanged compared to oligomers generated in absence of SUV (asyn/asyn129E: 112/147 monomers per oligomer) as determined by FIDA analysis. SIFT segment analysis further corroborated a reduced binding capacity of asyn129E oligomers as compared to asyn (figure 3 B). As indicated in figure 1 A, this analysis allows to depict the distribution and mixing ratio of bicolored aggregates. For the phosphorylation mimic asyn129E, the amount of bicolored particles (found in segments 4–15) is strongly reduced for both Al^3+^- and Fe^3+^-induced oligomers indicating strongly reduced membrane binding of these oligomers.

## Discussion

In the recent decade, the increasing knowledge of asyn aggregation behavior has shifted the scientific focus from the histopathologically visible fibrillar aggregates to small oligomer species. Dissecting the complex pathways of asyn oligomer formation and interactions of these oligomers with lipid membranes appears to be crucial to the understanding of synucleinopathies.

### Influence of phosphorylation-mimicking mutations on asyn oligomer formation

To date, both pro- [20]–[22] and anti-aggregatory effects [23], [24] of asyn phosphorylation and pseudophosphorylation have been observed *in vitro* and *in vivo*. In the present study, we applied fluorescently labeled asyn to investigate the influence of pseudophosphorylation at position Ser129 (asyn129E) on oligomer formation. In the absence of aggregation inducers, both asyn and asyn129E showed no spontaneous oligomer formation at nanomolar protein concentrations. The low concentrations applied in these experiments may account for the fact that this result is different from previous studies that reported increased aggregation of phosphorylated asyn, since it is known that high concentrations of asyn are required to enable spontaneous fibril formation.

Remarkably, the current study further demonstrates that asyn129E oligomer formation is largely increased as compared to wild-type protein upon coincubation with both Fe^3+^ and Al^3+^. The resulting oligomers did not differ in size compared to the unphosphorylated protein, with each oligomer containing approximately 115 to 160 monomers as determined by fluorescence intensity distribution analysis, which is in accordance with previous findings [6]. Interestingly, we recently demonstrated that phosphorylation of tau by Glycogen Synthase Kinase 3β enhances both metal-ion induced oligomer formation and coaggregation with asyn [33], [35]. The results presented here substantiate the hypothesis that phosphorylation facilitates metal-ion induced oligomer formation in amyloidogenic proteins at protein concentrations that likely resemble conditions in early states of disease. It was speculated that the positively charged metal ions facilitate protein interactions via the additional negative charges introduced to the protein structure through phosphorylation [36].

### Modeling phosphorylation at Ser129 has a mild influence on monomer binding to lipid membranes

**Figure 4:**
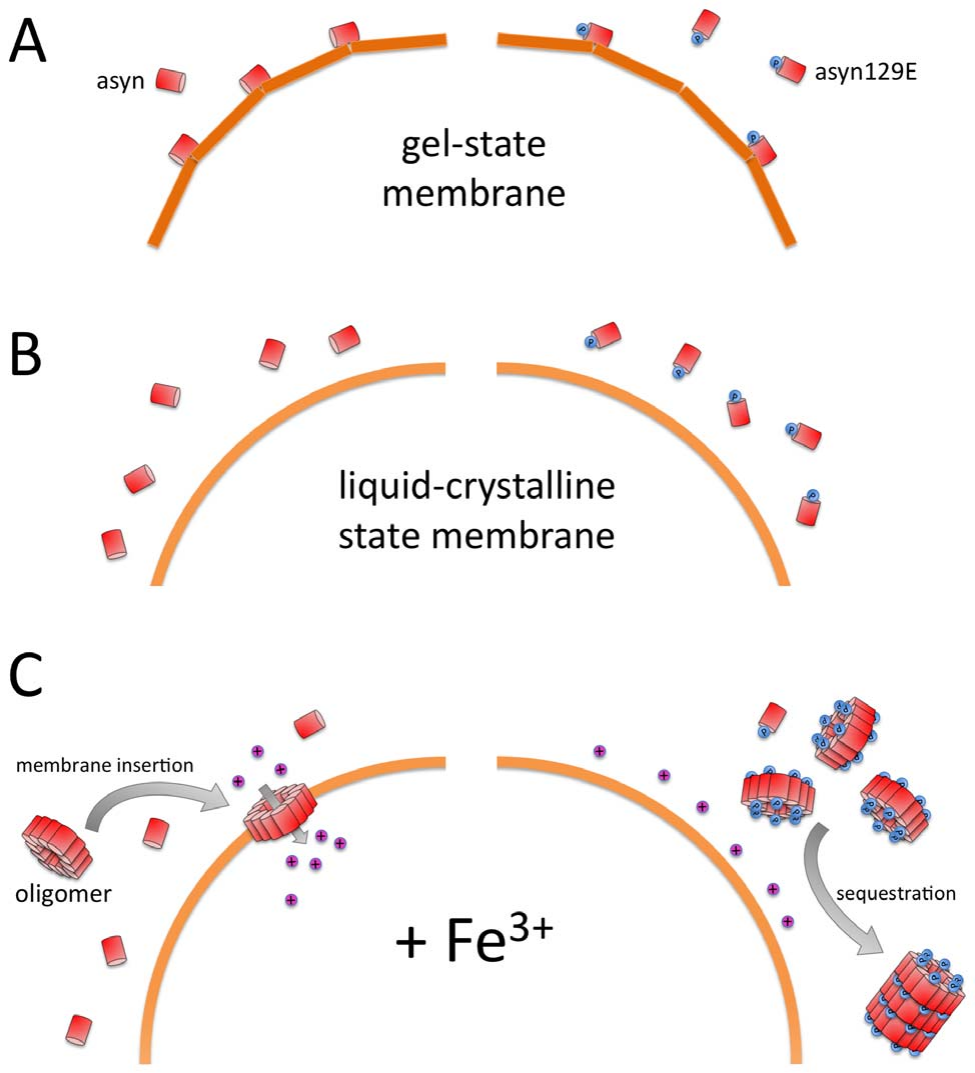
Schematic illustrating the influence of mimicking Ser129 phosphorylation on asyn membrane interactions. A. For stressed gel-state membranes (DPPC-SUV), mimicking phosphorylation at Ser129 mildly reduces asyn binding affinity. B. Both pseudophosphorylated and unphosphorylated asyn monomers show no affinity to membranes in the liquid-crystalline state (POPC). C. Fe^3+^ induced asyn oligomers show a high membrane affinity and potentially act as membrane pores. Pseudophosphorylation at Ser129 shows a differential influence on asyn aggregation and binding behaviour. While increasing oligomer formation in presence of trivalent metal-ions, Ser129 pseudophosphorylation inhibits membrane binding and may thus allow oligomer sequestration into larger aggregates such as fibrils.

Membrane binding of asyn is a complex interaction of various factors including lipid composition, membrane curvature, and posttranslational modifications of the protein [7], [37], [38]. It has previously been shown that while the N-terminus acts as the primary membrane binding region undergoing conformational changes, posttranslational modifications at the protein's C-terminus can alter membrane interactions [39]. Several lines of evidence indicate that asyn phosphorylation inhibits membrane binding. In both budding yeast and C. elegans, binding of asyn to plasma membranes is enhanced in the phosphorylation-deficient S129A mutation, while the phosphorylation mimic S129D shows decreased membrane binding [16], [25]. In our experiments, asyn pseudophosphorylation at Ser129 showed only a mild inhibitory effect on monomer binding to DPPC-SUV vesicles. Similar to wild-type asyn, no interactions with uncharged membranes in the liquid-crystalline state such as POPC-SUV were seen. This is in accordance with our previous findings that membrane interaction is dependent on the membrane surface configuration, requiring a negative surface charge or a stressed gel-phase to enable monomer binding [7] (figure 4). In small unilamellar vesicles consisting of DPPC as applied in the current study, the high membrane curvature leads to local defects in the vesicle membrane. It was demonstrated previously that monomeric asyn binds to these defects, thus leading to local reorganization of lipids [37], [40].

In summary, our observation of a mild inhibitory influence of Ser129 pseudophosphorylation on asyn monomer binding to DPPC-SUV further corroborates previous findings that phosphorylation inhibits membrane binding of asyn monomers, although the overall effect appears to be mild. Phosphorylation of monomeric asyn may thus be a physiological regulatory mechanism to adjust asyn membrane affinity.

### Modeling phosphorylation at Ser129 abolishes membrane binding of Fe^3+^ induced asyn oligomers

We have previously demonstrated that Fe^3+^ induced asyn oligomers exhibit a potentially pathological gain of function, acting as membrane pores on lipid surfaces [7], [8]. In a model system applying evaporation to generate asyn oligomers, a similar extent of membrane permeabilization was found, requiring a high proportion of negatively charged lipids to facilitate permeabilization [41]. In this model, an oligomer structure with a hydrophobic core composed of the N-terminus including the NAC domain was proposed, with the C-termini forming an outer shell with conformational heterogeneity [42], [43]. Our current findings indicate that phosphorylation at Ser129, while enhancing Fe^3+^ induced oligomer formation, may rescue membrane permeabilization by inhibiting oligomer binding to lipid surfaces. In our experiments, the high affinity of Fe^3+^ induced asyn oligomers was almost completely abolished by pseudophosphorylation. These results are surprising given that earlier studies demonstrated that interactions between asyn and lipid bilayers mainly occur through the N-terminal domain. Thus, our findings may point towards a potential role of the C-terminal domain in membrane interactions, as was demonstrated for other post-translational modifications [39]. Conversely, the effects observed could be due to structural differences between the ferric iron induced oligomers observed here and asyn oligomers generated by other means.

Of note, binding to POPC membranes was not limited to Fe^3+^ induced asyn oligomers, but appears to be a common hallmark of oligomers formed in presence of trivalent metal-ions. A similar, though less pronounced effect of asyn pseudophosphorylation was seen in Al^3+^ induced asyn oligomers, where vesicle binding of oligomers was reduced to <20%. While no significant difference in oligomer size was seen between asyn and asy129E, it is currently unknown whether mimicking phosphorylation alters the internal oligomer structure. The sequestration of potentially toxic oligomers from cellular membranes may act as a protective mechanism, possibly resulting in the formation of larger fibrillar asyn aggregates without toxic effects (also see figure 4).

In summary, our findings implicate a differential influence of asyn phosphorylation on aggregation behavior and membrane binding, as is illustrated in figure 4. Phosphorylation at Ser129 yields a mild inhibitory effect on monomer membrane binding. While it facilitates oligomer formation in presence of trivalent metal-ions, membrane binding of the resulting oligomers is strongly reduced. Thus, the current model suggests that the protective effect of asyn pseudophosphorylation recently seen in animal models is due to decreased membrane binding of asyn oligomers. Moreover, these results indicate that membrane binding is an essential mediator of neurotoxicity. The increased aggregation propensity of the phosphorylated protein may, in turn, lead to sequestration into large inert aggregates such as fibrillar deposits in form of Lewy bodies.

Importantly, these findings put a new complexion on experimental pharmaceutical interventions directed against POLO-2 kinase, which mediates asyn phosphorylation at Ser129 [44]. Given the potential neuroprotective effect of Ser129 phosphorylation, such interventions may not achieve relevant therapeutic effects.

## Supporting Information

Figure S1 **Addition of ferric iron does not result in quenching of Alexa/Bodipy fluorescent dyes.** Concentration series of ferric iron were added to different fluorescent dyes to investigate potential quenching effects. A/B. In a broad range of concentrations tested, Fe^3+^ does not alter particle brightness, diffusion time or fitted particle number of the fluorescent dye Alexa-647 as determined by FCS (A) and 1D-FIDA (B) analyses. C. Upon ligation to the non-amylodiogenic, asyn-specific antibody 15G7, no effect on Alexa-647 readout parameters is detectable in presence of 10 uM Fe^3+^. As demonstrated earlier, the aggregation-inducing effect of the metal ion appears to be specific to amyloidogenic proteins [35]. D. As observed for Alexa-647, no effect on readout parameters is detectable upon coincubation of Bodipy-PE and ferric iron in FIDA analysis. E. / F. Control experiment using asyn with point mutations to cysteine in positions 3, 81 and 140 and point-specific labeling with Alexa647 maleimide dyes. E. Oligomer sizes of Fe^3+^ induced asyn oligomers are comparable for all three point mutations and in accordance with randomly labeled wt asyn oligomers as demonstrated earlier [35]. F. No difference is seen in diffusion time of the different asyn mutants.
(TIF)
