# Supplementary figures and images for: Modelling Ser129 Phosphorylation Inhibits Membrane Binding of Pore-Forming Alpha-Synuclein Oligomers
Source: PLoS One. 2014 Jun 9;9(6):e98906. doi: 10.1371/journal.pone.0098906 (PMC4049638; doi:10.1371/journal.pone.0098906)

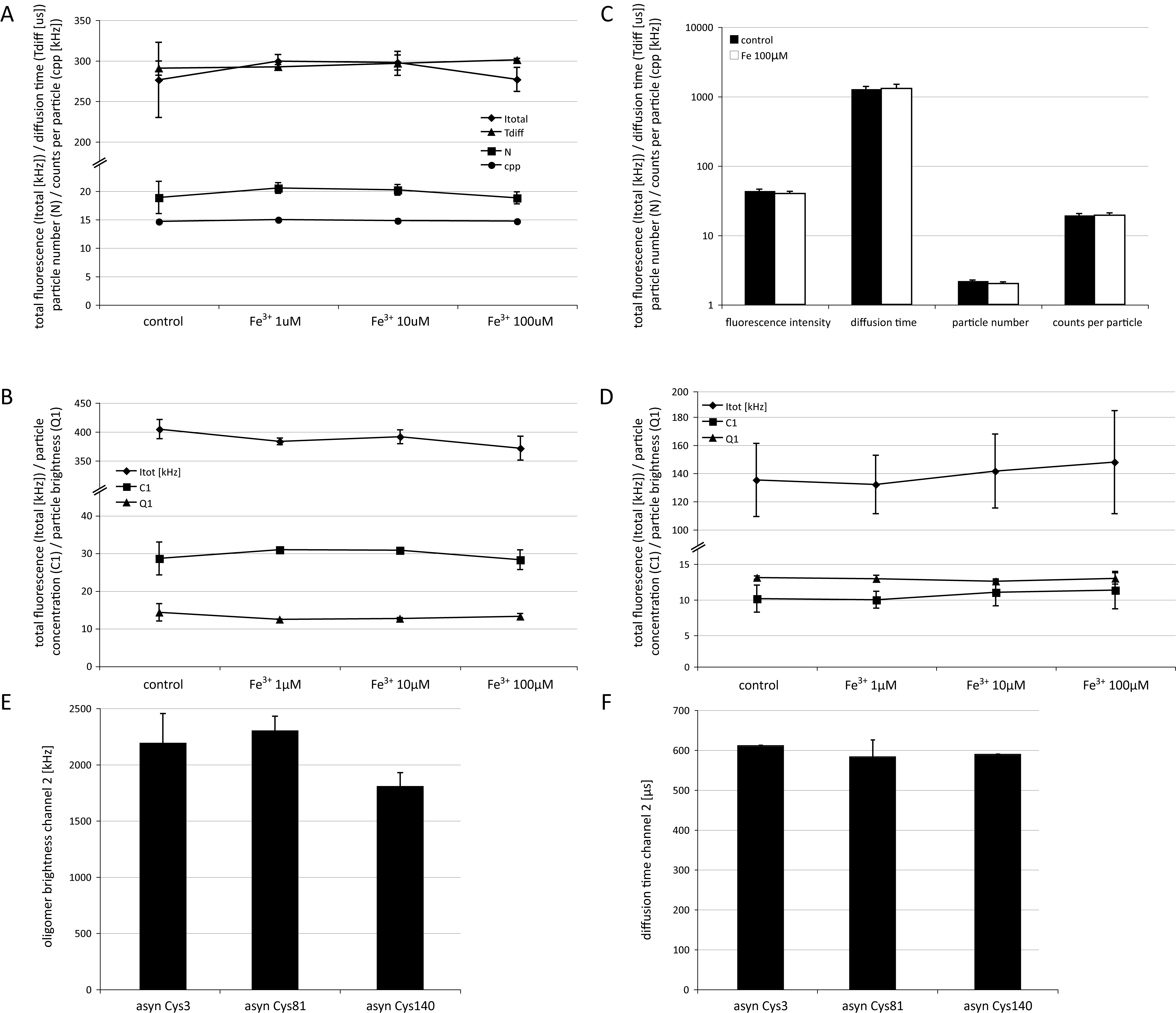

Supplement: Figure S1 — Addition of ferric iron does not result in quenching of Alexa/Bodipy fluorescent dyes. Concentration series of ferric iron were added to different fluorescent dyes to investigate potential quenching effects. A/B. In a broad range of concentrations tested, Fe3+ does not alter particle brightness, diffusion time or fitted particle number of the fluorescent dye Alexa-647 as determined by FCS (A) and 1D-FIDA (B) analyses. C. Upon ligation to the non-amylodiogenic, asyn-specific antibody 15G7, no effect on Alexa-647 readout parameters is detectable in presence of 10 uM Fe3+. As demonstrated earlier, the aggregation-inducing effect of the metal ion appears to be specific to amyloidogenic proteins [35]. D. As observed for Alexa-647, no effect on readout parameters is detectable upon coincubation of Bodipy-PE and ferric iron in FIDA analysis. E. / F. Control experiment using asyn with point mutations to cysteine in positions 3, 81 and 140 and point-specific labeling with Alexa647 maleimide dyes. E. Oligomer sizes of Fe3+ induced asyn oligomers are comparable for all three point mutations and in accordance with randomly labeled wt asyn oligomers as demonstrated earlier [35]. F. No difference is seen in diffusion time of the different asyn mutants. (TIF) [file pone.0098906.s001.tif]
